# Can the Combined Use of the Mirid Predator *Nesidiocoris tenuis* and a Braconid Larval Endoparasitoid *Dolichogenidea gelechiidivoris* Improve the Biological Control of *Tuta absoluta*?

**DOI:** 10.3390/insects12111004

**Published:** 2021-11-08

**Authors:** Pascal Osa Aigbedion-Atalor, Martin P. Hill, Pascal Mahukpe Ayelo, Shepard Ndlela, Myron P. Zalucki, Samira A. Mohamed

**Affiliations:** 1 International Centre of Insect Physiology and Ecology (icipe), Nairobi P.O. Box 30772-00100, Kenya; patalor@icipe.org (P.O.A.-A.); payelo@icipe.org (P.M.A.); sndlela@icipe.org (S.N.); 2 Centre for Biological Control, Department of Zoology and Entomology, Rhodes University, P.O. Box 94, Makhanda (Grahamstown) 6140, South Africa; m.hill@ru.ac.za; 3 Department of Zoology and Entomology, University of Pretoria, Private Bag X20, Hatfield 0028, Pretoria, South Africa; 4 School of Biological Sciences, The University of Queensland, Brisbane 4072, Australia; m.zalucki@uq.edu.au

**Keywords:** predator–parasitoid interactions, predatory mirid bug, *Apanteles gelechiidivoris*, South America tomato pinworm, biocontrol

## Abstract

**Simple Summary:**

Combining natural enemies may lead to synergistic, additive, or antagonistic effects on the control of insect pests. An investigation into the nature and outcome of the interaction between a generalist mirid predator, *Nesidiocoris tenuis*, and a specialist koinobiont larval endoparasitoid, *Dolichogenidea gelechiidivoris*, in the control of a co-shared host/prey, *Tuta absoluta*, was undertaken under laboratory conditions. We found that the presence of *N. tenuis* did not affect oviposition performance or progeny production by *D. gelechiidivoris*. When both natural enemies were combined, the efficacy in reducing *T. absoluta* populations was significantly higher than that of either natural enemy used alone. *Nesidiocoris tenuis* preferentially reduced the densities of *T. absoluta* eggs, while *D. gelechiidivoris* reduced the larval stages of the pest. The combined use of *N. tenuis* and *D. gelechiidivoris* could potentially help reduce the overall infestation level of *T. absoluta* in tomato agroecosystems.

**Abstract:**

The koinobiont solitary larval endoparasitoid *Dolichogenidea gelechiidivoris* (Marsh) (Syn.: *Apanteles gelechiidivoris*) (Hymenoptera: Braconidae) and the predatory bug *Nesidiocoris tenuis* (Reuter) (Hemiptera: Miridae) are important natural enemies of *Tuta absoluta* (Meyrick) (Lepidoptera: Gelechiidae), a serious pest of tomato. Although *N. tenuis* preferentially feeds on *T.* *absoluta* eggs, it is also recorded as a predator of first and second instar larval stages. *Dolichogenidea gelechiidivoris* preferentially seeks these early larval stages of *T. absoluta* for oviposition. The occurrence of intraguild predation between *N. tenuis* and *D. gelechiidivoris* and the consequences on the oviposition performance of *D. gelechiidivoris* were investigated in the laboratory. Regardless of the manner of introduction (i.e., the sequence of combinations with *D. gelechiidivoris*) or density (i.e., number of *N. tenuis* combined with *D. gelechiidivoris*), the presence of *N. tenuis* did not affect the oviposition performance of *D. gelechiidivoris* or the parasitoid’s progeny. Combination assays revealed that the efficacy of the combined use of *N. tenuis* and *D. gelechiidivoris* in controlling *T. absoluta* populations was significantly higher than that of either natural enemy alone. Our results highlight the potential of combining mirid predators and koinobiont larval endoparasitoids to control *T. absoluta*. The findings further contribute to data supporting the release of *D. gelechiidivoris* in tomato agroecosystems for the control of *T. absoluta* in Africa, where *N. tenuis* is widespread and abundant.

## 1. Introduction

The rationale for the use of multiple natural enemies in classical biological control has been justified by the assumption that one of the species released would establish and cause control, without concern for interaction effects [1]. Although some notable successes have been recorded [1,2], interactions between two or more natural enemies are not straightforward and the outcome may be counterproductive [3,4]. Interactions can be synergistic—the cumulative efficacy of both species being significantly higher than the efficacy of the two natural enemies when used separately [5]—or additive—when the combined use of both natural enemies is more effective and equal to the added efficacy by each species alone [6]. In other cases, interactions may be equivalent—when the combined impacts of both natural enemies are equal to the more effective of the two species [7]. Finally, interactions may be antagonistic—when the combined efficiency of two natural enemies is significantly less than the most effective species alone, resulting from adverse intraguild interactions [8,9]. These different possible outcomes indicate that the release of multiple natural enemies does not necessarily equate to success [1].

Intraguild predation occurs when one natural enemy species (intraguild predator) feeds on another (intraguild parasitoid), and it is the most researched intraguild interaction [10]. It could be direct (e.g., consumption of the intraguild parasitoid) or indirect (e.g., consumption of parasitized hosts and reduction in the oviposition performance of a parasitoid). The occurrence of intraguild predation between predators and parasitoids is generally linked to the inability of generalist predators to discriminate parasitised prey [11,12]. In other cases, it occurs as a result of the lack of discernment or avoidance of intraguild risks by parasitoids [13,14].

Modern approaches to biological control accentuate the need for improved evaluations, justifying the use of multiple agents [1,15,16]. Indeed, this approach can significantly reduce unnecessary releases of natural enemies and increase synergism or additive effects. When properly utilized, multiple agents can substantially increase the pests’ mortality [17]. The South American tomato pinworm, *Tuta absoluta* (Meyrick) (Lepidoptera: Gelechiidae), has been subjected to multiple natural enemies in the Palaearctic and Afrotropical regions since 2006 [16,18,19,20]. When no control measures are applied, *T. absoluta* can cause yield losses of 80–100% in tomatoes. Although the larvae—the destructive stage of the pest—can feed on fruits, they preferentially feed on the leaf tissues, resulting in the disruption of the host plant’s photosynthetic process [21,22]. Both in the native and invasion range, synthetic chemical insecticides are the primary management method for *T. absoluta* [19,21,23]. However, owing to its ability to rapidly evolve resistance to most insecticides as well as the deleterious ecological consequences associated with excessive use of synthetic insecticides [24], biological control encompassing the use of parasitoids, predators, and pathogens has been recommended, albeit in conjunction with other control methods [19,21,23,25].

Among the predators used for the biological control of *T. absoluta*, the zoophytophagous predatory bug *Nesidiocoris tenuis* (Reuter) (Hemiptera: Miridae) is one of the most important, owing to its high feeding capacity on eggs of *T. absoluta* [7,26,27]. This mirid is an omnivore, feeding on the early larval instars of the pest as well [26], and sometimes on tomato plants—in the absence of prey [28]. However, it prefers the eggs of *T. absoluta.* The parasitoid *Dolichogenidea gelechiidivoris* (Marsh) (Syn. *Apanteles gelechiidivoris*) (Hymenoptera: Braconidae) is a koinobiont solitary larval endoparasitoid of a few related gelechiid species such as *T. absoluta*, *Keiferia lycopersicella* (Walshingham), and *Phthorimaea operculella* (Zeller) [18,29]. *Dolichogenidea gelechiidivoris* is native to Peru, Colombia, Chile, and Spain [30,31]. This parasitoid wasp was imported into Africa in 2017 by the International Centre of Insect Physiology and Ecology (*icipe*), Kenya. It was recently released in three African countries (Ethiopia, Kenya, and Uganda) in the first classical biological control program against *T. absoluta* on the African continent. High parasitism rates of 57 to 77% of *T. absoluta* by *D. gelechiidivoris* in tomato fields have been reported across South America [32,33,34], as well as under quarantine laboratory conditions in Africa [18].

*Nesidiocoris tenuis* occurs in tomato agroecosystems in Africa, including the three countries where *D. gelechiidivoris* has been released [19,35,36]. So far, investigations into the nature of interactions between natural enemies of *T. absoluta* have been limited to egg parasitoids and predators belonging to the family Miridae or Nabidae [11,13,37]. Some studies have investigated interactions between idiobiont larval ectoparasitoids and mirid predators for the control of *T. absoluta* [10,38,39]. However, to the best of our knowledge, no study has tested the interactions between koinobiont larval endoparasitoids of *T. absoluta* and mirid predators. The feeding on first and second larval instars of *T. absoluta* by *N. tenuis* [26], the preferred oviposition host stages of *D. gelechiidivoris* [18], could be a source of mortality for the progeny of the parasitoid. Therefore, this study investigated the direct interactions between *N. tenuis* and the *D. gelechiidivoris*. We expected a negative interaction, meaning that, without the eggs of *T. absoluta*, *N. tenuis* would inflict significant intraguild predation on *D. gelechiidivoris* progeny within parasitized host larvae, thereby reducing the parasitoid’s progeny.

## 2. Materials and Methods

### 2.1. Plant Production

Tomato (cv. Moneymaker) plants were grown under a screen house at *icipe*, Nairobi, in plastic pots (10.5 cm diam., 14.5 cm high). Each pot contained about 2.5 kg of peat compost mixed with one hundred grams of N.P.K fertilizer (22:6:12, N:P:K) (Mea Ltd., Nairobi, Kenya) at planting. Tomato seedlings were transplanted at a density of two plants per pot and watered as needed. Five- to six-week-old potted plants were used in the experiments.

### 2.2. Insect Rearing

Colonies of *T. absoluta*, *D. gelechiidivoris*, and *N. tenuis* were reared and maintained in a level two laboratory (designed for newly-imported exotic natural enemies) in the quarantine facility of *icipe*, Nairobi, Kenya (1°17′08.844″ S, 36°49′12.108″ E). The environmental conditions in the quarantine facility were maintained at 25 ± 1 °C, 65 ± 5% RH, and 12L/12D photoperiod regime. Insects were kept in single sleeved PERSPEX^®^ (Jinbao, Yantai, China) cages (65 × 45 × 45 cm), containing four potted tomato plants for oviposition and feeding. Small streaks of 80% honey solution were smeared on the top sides of the cage to provide an additional food for the insects. 

#### 2.2.1. *Tuta absoluta* Colony

*Tuta absoluta* (eggs, larvae, and pupae) were collected from infested tomato plants in 10 different counties of Kenya (see Table S1). The infested tomato plants were placed in cages described above to allow eggs and larvae to develop into adults. Following emergence, adults of *T. absoluta* were aspirated into new cages of similar size and each containing tomato plants for oviposition. Eggs were allowed to develop and hatch into larvae, which were then used in the various experiments. 

#### 2.2.2. *Dolichogenidea gelechiidivoris* Colony

Two hundred cocoons of *D. gelechiidivoris* were received from the International Potato Centre (CIP), Peru (16°57′36.3″ N, 96°28′32.1″ W) in March 2017. The cocoons were placed in a single-sleeved PERSPEX^®^ cage (30 × 30 × 30 cm), and the parasitoid rearing was maintained at quarantine under 24 ± 4 °C, 65 ± 5% RH and 12/12 h L/D photoperiod regime, as described in a previous study [18]. Upon emergence, parasitoids were transferred into another cage and allowed to mate. The parasitoid wasps were offered tomato plants infested with early *T. absoluta* larvae for host parasitization for 48 h. Infested tomato plants were obtained by exposure of five- to six-week old plants to a cohort of *T. absoluta* moths for 3 days. New batches of plants with first and second instar larvae were provided to the wasps every 48 h. Thereafter, plants exposed to parasitism were incubated with addition of healthy tomato plants to ensure better development of the parasitized larvae until emergence of adults. Parasitoids were fed on 80% honey solution. Only parasitoid females (1-day-old) were used in the experiments.

#### 2.2.3. *Nesidiocoris tenuis* Colony

Adults of *N. tenuis* were collected from infested tomato plants in the same 10 counties in Kenya as *T. absoluta* (see Table S1). Adults of *N. tenuis* were placed in cages containing four potted tomato plants, as described for *T. absoluta*. In addition to the honey solution and water, non-viable frozen eggs of *Ephestia kuehniella* Zeller (Lepidoptera: Pyralidae) (Biotop, Livron-sur-Drôme, France) and commercial pollen (M. LACARTE, Terce) were provided to *N. tenuis* as a supplementary food source. Both males and females (2- to 5-day-old) were used in the experiments. Differentiation of sex was done based on the description by Kim et al. [40], where females possess an oval and rounded abdomen with an ovipositor, and are generally of larger size than males. Prior to the assays, these insects were deprived of prey, pollen, and honey for 24 h, and kept only on tomato plants.

### 2.3. Bioassays

#### 2.3.1. Experiment 1: Intraguild Predation by *Nesidiocoris tenuis* on *Dolichogenidea gelechiidivoris*

Under the same environmental conditions used for insect rearing above, nine treatments and three controls were used to assess the intraguild interaction between *N. tenuis* and *D. gelechiidivoris* on the larvae of *T. absoluta* (Table 1). For each treatment, a tomato leaflet with 20 *T. absoluta* first instar larvae and the petiole inserted in a moistened cotton wool ball to prevent early wilting was placed in a small, single-sleeved PERSPEX^®^ cage (6.5 × 12 × 12 cm), in which little streaks of 80% honey solution were smeared on the internal walls. The infestation was done by placing the first larval instars on the leaflet and allowing them 1 h during which 90–100% of the larvae were able to mine into the leaf tissues prior to the experiment.

The treatments (Table 1) included (T1): introduction of one *N. tenuis* adult (either male or female) before a female *D. gelechiidivoris*; for this, one naïve *N. tenuis* was placed in a PERSPEX^®^ cage (6.5 cm × 12 cm × 12 cm), containing a tomato leaflet with 20 first instar larvae of *T. absoluta* for 24 h. Following the removal of *N. tenuis*, a naïve mated female *D. gelechiidivoris* was then placed in the cage and removed after 24 h. Thereafter, dead and alive larvae were counted, and then the leaflet was retained in the cage for subsequent development of *T. absoluta* larvae, albeit with an intermittent re-supply of healthy tomato leaves. The mean number of dead *T. absoluta* larvae due to feeding by *N. tenuis* was calculated based on the differences between dead larvae in the control and treatments having only the predator. The same number of larvae, leaflets, experimental arena, exposure time (24 h for both *N. tenuis* and *D. gelechiidivoris*), and biological characteristics of both natural enemies described above were used for the other treatments (see Table 1 for further details).

During incubation, all treatments and controls were monitored daily, and the number of formed cocoons of moth and wasp was recorded, after which they were placed in a petri-dish for the emergence of *T. absoluta* and parasitoid adults, which were counted and recorded. Six replicates were analysed per treatment, using only replicates in which the natural enemies were recovered alive at the end of the experiment.

#### 2.3.2. Experiment 2: Efficacy of *Dolichogenidea gelechiidivoris* with and without *Nesidiocoris tenuis*

Under the same conditions described above, the effectiveness of *D. gelechiidivoris* and *N. tenuis* (both alone and in combination) in suppressing the survival of *T. absoluta* (egg to adult) was evaluated. Tomato plants each with five leaflets were exposed to *T. absoluta* for egg-laying in single sleeved PERSPEX^®^ cages (65 × 45 × 45 cm) for 24 h to obtain a uniform cohort of eggs. Thereafter, 60 eggs were retained on two opposite leaflets of the plant, while excess eggs were removed using a camel hairbrush and other plant leaflets excised. The leaflets bearing the eggs were carefully inserted into a plastic nalophan bag (used as test arena) and the proximal end of each bag was attached to the plant stem. The following treatments (natural enemies) were introduced into each bag for 24 h: (i) two *N. tenuis* adults alone (Nt2); (ii) one *D. gelechiidivoris* female alone (Dg)—here, first instar larvae had eclosed (5 days later) from the 60 eggs before the parasitoid was introduced; (iii) two *N. tenuis* adults and one *D. gelechiidivoris* female (Nt2Dg)—here, the *N. tenuis* were removed 24 h after introduction, and the *T. absoluta* eggs remaining after *N. tenuis* feeding were retained on the leaflet for 5 days allowing eclosion of first instar larvae prior to the introduction of the parasitoid; and (iv) control (C)—no natural enemies present.

After being exposed to the natural enemies, the infested leaflets were detached from the plant and the petioles were inserted into a moistened cotton wool ball to prevent early wilting. The leaflets were then placed into small PERSPEX^®^ cages (6.5 × 12 × 12 cm) for subsequent *T. absoluta* larval development, albeit with an intermittent re-supply of fresh and pest-free tomato leaves. The cages with the leaflets were kept in an insect growth chamber at a constant environmental condition of 25 ± 1 °C, 65 ± 5% RH, and photoperiod regime of 12L/12D. The experiment was monitored daily. Cocoons and pupae formed were then placed in petri-dishes for the emergence of the parasitoid and *T. absoluta* adults, which were counted and recorded. The experiment was replicated six times.

### 2.4. Statistical Analyses

All analyses were performed in R. version 4.0.0 [41]. Owing to the binary nature of the data from *T. absoluta* mortality (dead versus alive), we used the generalised linear model (GLM), with binomial distribution and log link function, to test whether this variable was influenced by the different *N. tenuis* densities. We established the significance of the model using analysis of deviance (with Chi-square test). Data from the number of *D. gelechiidivoris* cocoons formed and total adults emerged, and the number of parasitoid females emerged, as well as the number of emerged *T. absoluta* adults from each treatment, were normally distributed (Shapiro–Wilks tests: *p* > 0.05), and their variance was homogenous (Bartlett’s test: *p* > 0.05). Therefore, we used the analysis of variance (ANOVA) for treatment effects with these data. Data on the efficacy of *D. gelechiidivoris* and *N. tenuis* both alone and in combination on the population density of *T. absoluta* followed a normal distribution model and their variance was similar. So, we performed ANOVA followed by the Student–Newman–Keuls (SNK) post-hoc tests to separate the means at α = 0.05.

## 3. Results

### 3.1. Experiment 1: Intraguild Predation by Nesidiocoris tenuis on Dolichogenidea gelechiidivoris

**Figure 1:**
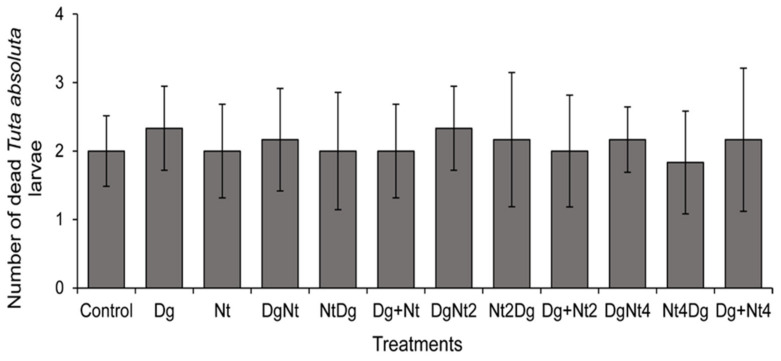
Mean (±SE) number of dead *Tuta absoluta* larvae following their exposure to *Nesidiocoris tenuis* (Nt) adults in varying density combinations (1, 2, and 4) for 24 h and a single *Dolichogenidea gelechiidivoris* (Dg) female for 24 h, either alone or in a sequential or simultaneous exposition; i.e., (DgNt) = 20 host larvae exposed first to Dg and then transferred to Nt; (NtDg) = 20 host larvae exposed first to Nt and then transferred to Dg; and (Dg + Nt) = simultaneous introduction of the biocontrol agents. No significant differences were found between the treatments.

With or without *D. gelechiidivoris*, *N. tenuis* host larval feeding was generally low (0.61 ± 0.2 larvae in 24 h) and not significant (GLM, χ^2^ = 70.852, df = 11, *p* = 0.910) (Figure 1).

**Figure 2:**
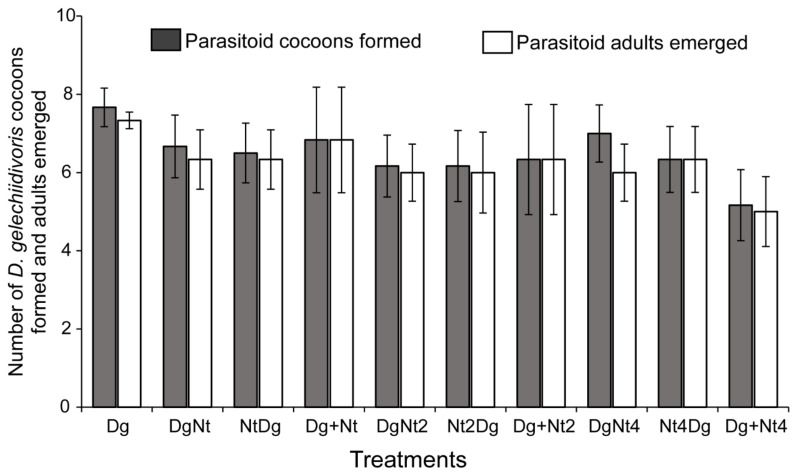
Mean (±SE) number of *Dolichogenidea gelechiidivoris* (Dg) cocoons formed and adults emerged in each of the treatments following a 24 h host larval predation exposure time to *Nesidiocoris tenuis* (Nt) in varying densities (1, 2, and 4) and in combination with a female *D. gelechiidivoris* for 24 h, in a sequential order of exposition; i.e., (DgNt) = 20 host larvae exposed first to Dg and then transferred to Nt; (NtDg) = 20 host larvae exposed first to Nt and then transferred to Dg, or simultaneous introduction (Dg + Nt) of the biocontrol agents. No significant differences were found between the treatments.

**Figure 3:**
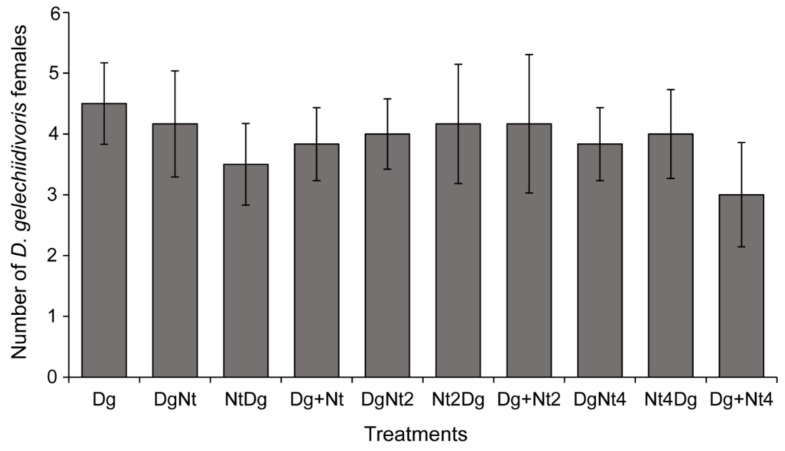
Mean (±SE) number of female *Dolichogenidea gelechiidivoris* (Dg) that eclosed from each of the treatments following a 24 h host larval predation exposure time to *Nesidiocoris tenuis* (Nt) in varying densities (1, 2, and 4) and in combination with a female *D. gelechiidivoris* for 24 h, in sequential order of introduction; i.e., (DgNt) = 20 host larvae exposed first to Dg and then transferred to Nt; (NtDg) = 20 host larvae exposed first to Nt and then transferred to Dg, or simultaneous introduction (Dg + Nt) of the biocontrol agents. No significant differences were found between the treatments.

In either simultaneous or sequential release, host larval feeding by *N. tenuis* did not affect the oviposition performance of *D. gelechiidivoris* and the parasitoid’s progeny. *Nesidiocoris tenuis* did not discriminate between parasitized and unparasitized *T. absoluta* larvae. This was reflected in the number of *D. gelechiidivoris* cocoons formed (F_9,50_ = 0.59, *p* = 0.861) (Figure 2), as well as in the number of parasitoid adults that emerged, which did not vary among the different treatments (F_9,50_ = 0.41, *p* < 0.92) (Figure 2). Similarly, no significant differences were recorded in the number of *D. gelechiidivoris* females that eclosed in each of the treatments (F_9,50_ = 0.32, *p* = 0.966) (Figure 3).

### 3.2. Experiment 2: Efficacy of Dolichogenidea gelechiidivoris on Tuta absoluta with and without Nesidiocoris tenuis

**Figure 4:**
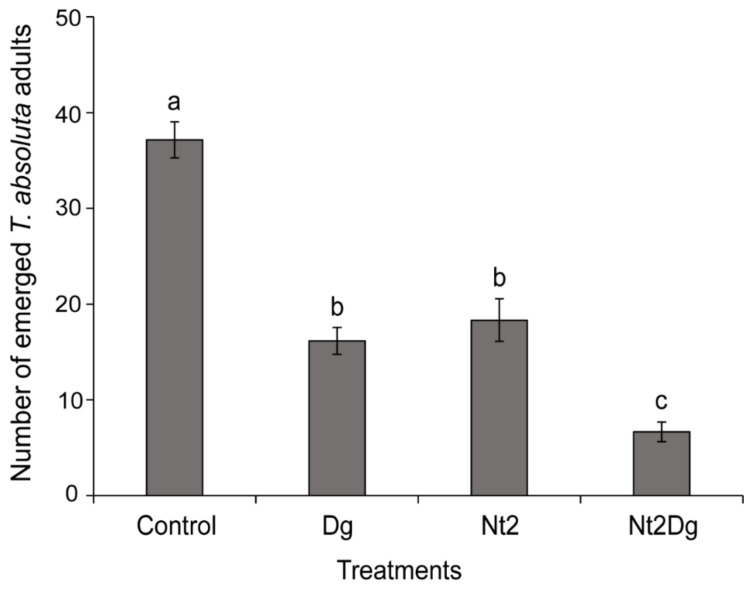
Mean (±SE) number of *Tuta absoluta* adults that emerged from each experimental treatment. Control = no natural enemy present during egg and larval development; Nt2 = two *Nesidiocoris tenuis* (a male and a female) present at host egg stage; Dg = one parasitoid *Dolichogenidea gelechiidivoris* female present at host larval stage; and Nt2Dg = two *N. tenuis* (a male and a female) exposed at host egg stage, then one *D. gelechiidivoris* female exposed at host larval stage. Bars are means (±SE) and different lowercase letters, following ANOVA, depict significant difference (SNK test *p* < 0.05).

*Dolichogenidea gelechiidivoris*, *N. tenuis*, and the combination of both natural enemies significantly reduced the number of *T. absoluta* adults that eclosed (F_3,20_ = 56.51 *p* < 0.001). Both natural enemies had a similar efficacy on the host when acting alone on their preferred host stage, compared with the control (Figure 4). However, when in combination (i.e., *N. tenuis* exposed to *T. absoluta* eggs, then *D. gelechiidivoris* exposed to hatching larvae), their combined efficacy significantly reduced *T. absoluta* adult eclosion compared with when either biological control agent acted alone (F_2,15_ = 14.47 *p* < 0.001) (Figure 4).

## 4. Discussion

Regardless of *N. tenuis* density and combination sequence with *D gelechiidivoris*, *N. tenuis* did not affect the oviposition performance of *D. gelechiidivoris* and the number of the parasitoid’s progeny. These findings thus fail to support our hypothesis of an inhibitory interaction between both natural enemies and suggest that the predator *N. tenuis* does not inflict intraguild predation on the parasitoid *D. gelechiidivoris*. However, the absence of empirical evidence of intraguild predation does not necessarily mean that *N. tenuis* discriminates between unparasitized *T. absoluta* larvae and those parasitized by *D. gelechiidivoris*. This is because we did not analyse the gut contents of the predator. Nevertheless, it is clear that *D. gelechiidivoris* does not suffer significant intraguild predation from *N. tenuis* in the laboratory environmental conditions, mainly because of the little *T. absoluta* larval feeding by *N. tenuis*. Low feeding capacity of the predator *N. tenuis* on *T. absoluta* larvae—about two larvae per day, compared with eggs, about 30 eggs per day—has been reported [26]. We found that larvae in mines were rarely killed; less than two larvae were eaten daily by *N. tenuis* in our experiments. Unlike the study of Urbaneja et al. [26], done under complete insect starvation (i.e., no prey and no plant offered), *N. tenuis* were offered plants in our study to simulate natural field conditions and larvae were in mines.

The prey feeding capacity of the predator *N. tenuis* is affected by factors such as sex of the predator, starvation, tomato cultivar, and trichome density [42,43], as well as genetic variation in *N. tenuis* [44]. On the other hand, as *D. gelechiidivoris* is a specialist endoparasitoid, we cannot rule out the possibility that the parasitoid avoided ovipositing into *T. absoluta* larvae that were punctured or explored by the predator, as a strategy to avoid the risk of intraguild predation. These findings suggest that *N. tenuis* can co-exist with *D. gelechiidivoris* in tomato agroecosystems without compromising the parasitoid’s progeny. However, field studies over a long-term period may provide more insights into the interactions between these two natural enemies. We would expect the predator to reduce the numbers of eggs, and hence the numbers of larval. Plant factors also impact larval survival [45], but a parasitoid that attacks early larval stages will reduce the numbers even further.

To the best of our knowledge, no previous studies have reported interactions between mirid predators and koinobiont larval endoparasitoids of *T. absoluta*. Chailleux et al. [39] reported co-existence between the mirid predator *Macrolophus pygmaeus* Rambur (Hemiptera: Miridae) and the idiobiont larval ectoparasitoid *Stenomesius* *japonicus* (Ashmead) (Hymenoptera: Eulophidae), but found that the predator affected the parasitoid’s population dynamics. Idiobiont parasitoids that paralyze their host larvae, making them immobile and unable react to aggression, are more vulnerable to intraguild predation than koinobiont parasitoids [46]. The strategies adopted and exploited by koinobiont endoparasitoids in limiting intraguild predation include hiding, restricted foraging and feeding, modification of the chemical stimuli, and aggressive defensive behaviour by the parasitized host larvae [46,47].

The efficacy trials showed that both *N. tenuis* and *D. gelechiidivoris* were effective in reducing the population density of *T. absoluta*. However, the combined efficacy of both natural enemies was more effective than either agent alone, indicating a potential synergism. The use of multiple natural enemies to control a pest in instances where they demonstrate synergistic or additive interactions have been recommended for biological control of crop pests [1,11,17,38,39]. Our study highlights the potential of combining koinobiont larval endoparasitoids such as *D. gelechiidivoris* with the predator *N. tenuis* for increased biological control of *T. absoluta*. Previous studies reporting interactions between idiobiont larval ectoparasitoids and generalist omnivorous mirid predators for the control of *T. absoluta* have indicated that, even though idiobiont larval ectoparasitoids suffer intraguild predation by mirid predators [10], the outcome is not always negative for the control of the target pest. This is exemplified by the combine use of *N. tenuis* and the idiobiont larval ectoparasitoid *Necremus tutae* (Walker) (Hymenoptera: Eulophidae); the combined use of both natural enemies reduced the population of *T. absolua* in greenhouses more than either of the two used alone [38]. Chailleux et al. [39] also documented that the predator *M. pygmaeus* and the idiobiont larval ectoparasitoid *S. japonicus* show complementary functional traits, resulting in a higher control of *T. absoluta* than when either natural enemy occurs alone. Similar synergistic or additive effects between eggs parasitoids and generalist omnivorous predators of *T. absoluta* have been reported. Although intraguild predation by *M. pygmaeus* was inflicted on *Trichogramma achaeae* Nagaraja and Nagarkatti (Hymenoptera: Trichogrammatidae), the risk of the intraguild predation was dependent on the larval developmental stages of *Trichogramma achaeae* in *T. absoluta* eggs, and the combination of both natural enemies was shown to be more effective for the control of *T. absoluta* when compared with the efficacy of the predator alone, thus suggesting an additive interaction between both natural enemies [11].

It has been shown that some generalist mirid egg predators can discern and avoid eggs that are parasitized, as reflected in the preference for unparasitized or newly parasitized *T. absoluta* eggs over old parasitized eggs by *M.* *pygmaeus* [11] and *N. tenuis* [48], as well as *Nabis pseudoferus* Remane (Hemiptera: Nabidae) [37]. Similarly, the predator *Dicyphus hesperus* (Knight) (Hemiptera: Miridae) also avoids parasitized *Bactericera cockerelli* (Šulc) (Hemiptera: Triozidae) nymphs having late instars of the parasitoid *Tamarixia triozae* (Burks) (Hymenoptera: Eulophidae) [49]. In our experiments, the 24 h exposure of host larvae to parasitism by *D. gelechiidivoris* was not long enough to allow parasitized hosts to reach late instars, suggesting that physiological changes in the host were not much to enable *N. tenuis* to discriminate between parasitized and unparasitized *T. absoluta* larvae.

In contrast to reports highlighting synergistic or additive interactions between some egg parasitoids and *N. tenuis,* there have been reports of equivalent interactions (i.e., neutral effects do not improve or reduce control) [7] and, in some cases, antagonistic interactions between *N. tenuis* and some eggs parasitoids of *T. absoluta* [9,50]. Specifically, Mirhosseini et al. [7] emphasized that releases of *Trichogramma brassicae* Bezdenko (Hymenoptera: Trichogrammatidae) together with *N. tenuis* resulted in 36% pest reduction compared with 40% pest reduction when *N. tenuis* is used alone. As egg parasitoids and mirid predators share the same stage of host/prey, perhaps egg parasitoids are more susceptible to intraguild predation by *M. pygmaeus* and *N. tenuis* than larval parasitoids [11,39]. Therefore, the combination of mirid predators with egg parasitoids should be done with caution.

Several indigenous natural enemies such as the larval parasitoids: *Bracon nigricans* Szépligeti (Hymenoptera: Braconidae), *Dolichogenidea appellater* (Telenga) (Hymenoptera: Braconidae), *Necremnus tutae* Ribes, and Bernardo (Hymenoptera: Eulophidae); as well as the egg parasitoids: *Trichogramma bourarachae* Pintureau and Babault, *Trichogramma cacoeciae* (Marchal) (Hymenoptera: Trichogrammatidae); and the egg predators: *M. pygmaeus*, *N. tenuis*, and *Rhynocoris segmentarius* (Germar) (Hemiptera: Reduvidae), have been reported as biological control agents of *T. absoluta* in several African countries [19,37,51]. *Dolichogenidea gelechiidivoris* has been released in three of these countries and releases in other countries are planned. However, very few studies evaluating the interactions between some of these natural enemies have been conducted [19]. It becomes rather crucial that further studies investigating the outcome of the interactions between these natural enemies be undertaken to improve our understanding of their impacts on the population density of *T. absoluta* in tomato agroecosystems.

## 5. Conclusions

In summary, augmentative releases of *N. tenuis* and *D. gelechiidivoris* could potentially help reduce the infestation levels of *T. absoluta* because, in a tomato agroecosystem, all host life stages of the pest usually occur, albeit in varying densities, thus indicating that the host life-stage preferred by each natural enemy would always be present, thereby limiting the outcome of predation on *D. gelechiidivoris*-parasitized *T. absoluta* larvae. Differential niche exploitation of oviposition and feeding resources have been theoretically and experimentally shown to promote coexistence between species of natural enemies in nature [52,53]. We anticipate that *N. tenuis* would preferentially feed on, and reduce densities of, *T. absoluta* eggs, while *D. gelechiidivoris* will parasitize and reduce the larval stages of the pest, consequently reducing the overall infestation level of the pest, and this could further alleviate the pest burden on tomato growers when integrated with other pest management approaches.

## Figures and Tables

**Table 1:** Treatment structure used in assessing the interaction between varying numbers of *Nesidiocoris tenuis* (Nt) adults and one *Dolichogenidea gelechiidivoris* (Dg) female for 24 h on the larval survival and development of *Tuta absoluta*.

Treatment	Delineation
(T1) Nt1Dg	One *N. tenuis* adult (either male or female) introduced before a female *D. gelechiidivoris*
(T2) Nt2Dg	Two *N. tenuis* adults (1 male and 1 female) introduced before a female *D. gelechiidivoris*
(T3) Nt4Dg	Four *N. tenuis* adults (2 males and 2 females) introduced before a female *D. gelechiidivoris*
(T4) DgNt1	A female *D. gelechiidivoris* introduced before one *N. tenuis* adult
(T5) DgNt2	A female *D. gelechiidivoris* introduced before two *N. tenuis* adults
(T6) DgNt4	A female *D. gelechiidivoris* introduced before four *N. tenuis* adults
(T7) Dg + Nt1	Simultaneous introduction of both natural enemies (one *N. tenuis* adult and one *D. gelechiidivoris* female)
(T8) Dg + Nt2	Simultaneous introduction of both natural enemies (two *N. tenuis* adults and one *D. gelechiidivoris*)
(T9) Dg + Nt4	Simultaneous introduction of both natural enemies (four *N. tenuis* adults and one *D. gelechiidivoris* female)
(T10) Dg	One *D. gelechiidivoris* introduced alone
(T11) Nt	Four *N. tenuis* adults introduced alone
(T12) Control	No natural enemy present

Numerals appended to “Nt” indicate the number of *N. tenuis* used for each assay. Note that T11: four *N. tenuis* adults alone with a tomato leaflet with 20 first instar larvae, served as control for mortality owing to feeding by *N. tenuis*; while T12: a tomato leaflet with 20 *T. absoluta* first instar larvae without the natural enemies, served as control for *Tuta absoluta* larval natural mortality.

## Data Availability

All datasets associated with this manuscript have been submitted to Dryad and are available for public use with the DOI https://datadryad.org/stash/share/19FnCZdUETKvBFkx8HBzemBI6l2NaX9ckwXp_BiKuxk (accessed on 9 September 2021).

## Supplementary Materials

The following are available online at https://www.mdpi.com/article/10.3390/insects12111004/s1, Table S1: Counties of Kenya where *Tuta absoluta* was collected to maintain the genomic pool of the established laboratory colony.
