# Supplementary material for: Can the Combined Use of the Mirid Predator Nesidiocoris tenuis and a Braconid Larval Endoparasitoid Dolichogenidea gelechiidivoris Improve the Biological Control of Tuta absoluta?
Source: Insects. 2021 Nov 8;12(11):1004. doi: 10.3390/insects12111004 (PMC8621560; doi:10.3390/insects12111004)
Supplement: Supplementary file 1 [file insects-12-01004-s001.zip › insects-1406149-supplementary.pdf]

Table S1 Counties of Kenya where *Tuta absoluta* was collected to maintain the genomic pool of the established laboratory colony

County	GPS coordinates	Number of sites sampled
Kajiado	1° 50' 30.984" S 36° 47' 21.3648" E	14
Kwale	4° 10' 31.116" S 39° 27' 11.952" E	7
Limuru	1° 6' 33.66" S 36° 38' 35.592" E	11
Machakos	1° 31' 6.348" S 37° 16' 0.8364" E	13
Meru	0° 2' 47.004" N 37° 39' 9.8928" E	8
Nakuru	0° 16' 53.364" S 36° 4' 42.3084" E	15
Nyeri	0° 25' 16.14" S 36° 56' 57.8724" E	2
Siaya	0° 3' 44.568" N 34° 17' 20.616" E	1
Taita Taveta	3° 23' 31.92" S 37° 40' 26.832" E	12
Mwea	1° 41' 47.04" S 37° 25' 2.712" E	5
